# Risk of Neurodevelopmental Disorders and Paternal Use of Valproate During Spermatogenesis

**DOI:** 10.1001/jamanetworkopen.2025.12139

**Published:** 2025-05-22

**Authors:** Jakob Christensen, Betina B. Trabjerg, Julie Werenberg Dreier

**Affiliations:** 1 Department of Clinical Medicine, Aarhus University, Aarhus, Denmark; 2 Department of Neurology, Aarhus University Hospital, Aarhus, Denmark; 3 National Centre for Register-Based Research, School of Business and Social Sciences, Aarhus University, Aarhus, Denmark; 4 Centre for Integrated Register-Based Research (CIRRAU), Aarhus University, Aarhus, Denmark

## Abstract

This cohort study assesses associations of paternal use of valproate with neurodevelopmental disorders in children.

## Introduction

In January 2024, the European Medicines Agency (EMA) recommended precautionary measures for the treatment of male patients with valproate.^1^ This followed an observational study report prepared by the contract research organization IQVIA, reporting a potential risk of neurodevelopmental disorders (NDDs) in children born to males treated with valproate during spermatogenesis.^1,2^ In a subsequent study based on a subset of the same data, we were unable to replicate the IQVIA study results.^3^ However, at the time when our study was accepted for publication (March 31, 2024), limited information was available about the IQVIA study, and a detailed online report was only published on May 17, 2024.^4^ To address the conflicting results between the 2 studies,^3,2^ we present additional analyses of our data material with further alignments to the IQVIA study.^2^

## Methods

This cohort study was approved by the Danish Data Protection Agency and did not require ethical review board approval or informed consent according to Danish law. The reporting followed the STROBE guideline. We identified all singleton children born alive in Denmark between 1997 and 2017. We excluded children of mothers who had undergone in vitro fertilization, with unknown or unrealistic gestational age, and without paternal link (98 670 children), leaving a population of 1 180 308 children. Paternal monotherapy exposure to valproate, lamotrigine, and levetiracetam was defined as filling 1 or more prescriptions for only 1 of these medications from 120 days before the first day of the last menstrual period (LMP) to 14 days after the LMP. Additionally, we conducted a sensitivity analysis with a restricted exposure window from 60 days before to 14 days after the LMP. Children were followed-up from age 1 year until December 31, 2018, for diagnoses of NDDs. We used Cox regression to estimate adjusted hazard ratios (aHRs) of NDDs comparing children exposed to valproate monotherapy with children exposed to lamotrigine or levetiracetam monotherapy, adjusted for relevant confounders (eTables 1-5 in Supplement 1).^3^ A range of analyses were performed to explore the impact of methodological choices and definitions used in the IQVIA study^2^: excluding children of parents with NDDs or congenital malformations, excluding children of mothers with epilepsy; excluding children with epilepsy, rerunning analyses without adjustment for parental epilepsy and education, and stopping follow-up at age 12 years. Data were analyzed in February 2025 using SAS version 9.4 (SAS Institute). Statistical significance was a 2-sided *P* < .05.

## Results

In the overall analysis based on 961 children (501 male [52.1%]; median [IQR] age at the end of follow-up, 10.6 [5.4-15.0] years) exposed to valproate monotherapy and 1401 children (725 male [51.8%]; median [IQR] age at end of follow-up, 5.5 [2.7-9.3] years) exposed to lamotrigine or levetiracetam monotherapy, paternal valproate exposure was not associated with an increased risk of NDDs (composite end point of all NDD aHR, 1.02; 95% CI, 0.67-1.54). This finding remained robust across analyses of specific NDDs, analyses of valproate dose, analyses accounting for time trends, analyses allowing for polytherapy, analyses expanding the definition of NDDs, analyses restricted to fathers with epilepsy, and analyses with a restricted exposure window (Table 1). In analyses restricted to fathers with epilepsy of unknown underlying cause, the aHR was 2.48 (95% CI, 1.13-5.44)^5^; however, the association was no longer significant in analyses matched on birth year (aHR, 2.33; 95% CI, 1.00-5.44). Analyses undertaken to further examine the definitions used in the IQVIA study^2^ revealed no increased risk of NND associated with valproate exposure (Table 2).

**Table 1.** Risk of NDDs in Children of Fathers Who Used Valproate Compared With Children of Fathers Who Used Lamotrigine or Levetiracetam During Spermatogenesis

Analysis and comparison groups	Exposed, No.	Follow-up time, median (IQR), y	NDD cases, No.	IR/1000 person-years	Risk of NDD, HR (95% CI)	
					Unadjusted	Adjusted
Main analysis						
Composite end point of all NDDs^a^						
Lamotrigine or levetiracetam	1401	5.5 (2.7-9.3)	50	5.6	1.00 [Reference]	1.00 [Reference]
Valproate	961	10.6 (5.4-15.0)	67	6.7	0.99 (0.68-1.44)	1.02 (0.67-1.54)
Intellectual disability^b^						
Lamotrigine or levetiracetam	1401	5.6 (2.7-9.5)	7	0.8	1.00 [Reference]	1.00 [Reference]
Valproate	961	11.1 (5.8-15.4)	21	2.1	2.29 (0.96-5.46)	1.85 (0.72-4.76)
Disorders of psychological development^c^						
Lamotrigine or levetiracetam	1401	5.5 (2.7-9.5)	11	1.2	1.00 [Reference]	1.00 [Reference]
Valproate	961	11.2 (5.7-15.5)	14	1.4	1.01 (0.45-2.25)	0.92 (0.38-2.24)
Autism spectrum disorder^d^						
Lamotrigine or levetiracetam	1401	5.5 (2.7-9.4)	31	3.5	1.00 [Reference]	1.00 [Reference]
Valproate	961	11.1 (5.7-15.4)	24	2.3	0.54 (0.31-0.93)	0.69 (0.37-1.28)
ADHD^e^						
Lamotrigine or levetiracetam	1401	5.5 (2.7-9.4)	27	3.0	1.00 [Reference]	1.00 [Reference]
Valproate	961	11.0 (5.6-15.4)	26	2.6	0.69 (0.40-1.19)	0.60 (0.33-1.12)
Secondary analyses						
Lamotrigine or levetiracetam	1401	5.5 (2.7-9.3)	50	5.6	1.00 [Reference]	1.00 [Reference]
Valproate						
High dose	490	11.0 (6.4-15.4)	33	6.3	0.92 (0.59-1.43)	0.99 (0.61-1.63)
Low dose	471	10.1 (4.6-14.6)	34	7.2	1.08 (0.69-1.68)	1.04 (0.64-1.67)
Accounting for calendar trends (matched on birth year)^f^						
Lamotrigine or levetiracetam	715	8.4 (4.2-12.1)	39	6.6	1.00 [Reference]	1.00 [Reference]
Valproate	715	8.3 (4.2-11.9)	41	7.2	1.07 (0.69-1.65)	1.09 (0.68-1.75)
Allowing for polytherapy and monotherapy in both groups^g^						
Lamotrigine or levetiracetam	1710	6.0 (2.8-10.0)	65	5.6	1.00 [Reference]	1.00 [Reference]
Valproate	1252	10.4 (5.3-14.9)	84	6.6	1.01 (0.73-1.41)	1.06 (0.74-1.50)
Including ADHD medication in definition of NDD						
Lamotrigine or levetiracetam	1401	5.4 (2.7-9.2)	55	6.2	1.00 [Reference]	1.00 [Reference]
Valproate	961	10.5 (5.4-15.0)	72	7.3	0.98 (0.68-1.40)	0.98 (0.66-1.45)
Restricted to fathers with epilepsy						
Lamotrigine or levetiracetam	737	6.4 (3.0-10.7)	20	3.8	1.00 [Reference]	1.00 [Reference]
Valproate	740	10.9 (6.4-15.1)	56	7.1	1.60 (0.96-2.69)	1.42 (0.82-2.46)
Restricted to fathers with epilepsy, matched on birth year						
Lamotrigine or levetiracetam	511	8.5 (4.7-12.1)	NA^h^	4.5	1.00 [Reference]	1.00 [Reference]
Valproate	511	8.5 (4.5-12.0)	24	5.8	1.42 (0.76-2.64)	1.14 (0.59-2.21)
Restricted to fathers with epilepsy of unknown underlying cause						
Lamotrigine or levetiracetam	478	7.0 (3.3-11.2)	8	2.2	1.00 [Reference]	1.00 [Reference]
Valproate	593	11.4 (6.9-15.4)	50	7.7	3.04 (1.43-6.44)	2.48 (1.13-5.44)
Restricted to fathers with epilepsy of unknown underlying cause, matched on birth year						
Lamotrigine or levetiracetam	362	8.5 (4.5-12.3)	8	2.6	1.00 [Reference]	1.00 [Reference]
Valproate	362	8.2 (4.4-11.9)	24	7.1	3.09 (1.39-6.89)	2.33 (1.00-5.44)

Abbreviations: ADHD, attention-deficit/hyperactivity disorder; HR, hazard ratio; IR, incidence rate; NA, not applicable; NDD, neurodevelopmental disorder.

^a^ In sensitivity analysis with a restricted exposure window from 60 days before to 14 after the first day of the last menstrual period, valproate exposure was not associated with NDD risk (adjusted HR [aHR], 0.96; 95% CI, 0.62-1.49). In analyses with follow-up time split in 2 periods, valproate exposure was not associated with NDD risk in either early (aHR, 1.30; 95% CI, 0.66-2.57) or late (aHR, 0.89; 95% CI, 0.52-1.50) follow-up periods.

^b^ *International Statistical Classification of Diseases and Related Health Problems, Tenth Revision (ICD-10) *codes F70-79.

^c^ *ICD-10* codes F80-83.

^d^ *ICD-10* code F84 (excluding F84.2-F84.4).

^e^ *ICD-10* codes F90.0 and F98.8.

^f^ Equal number of exposed and unexposed per birth year.

^g^ Children where the father filled prescriptions for valproate in combination with lamotrigine and/or levetiracetam were only included in the valproate group.

^h^ Differences less than 5 cannot be reported for data privacy reasons.

**Table 2.** Analyses Undertaken to Examine Definitions Used in the IQVIA Study^2^

Comparison	Exposed, No.	Follow-up time, median (IQR), y	NDD cases, No.	IR/1000 person-years	Risk of NDD, HR (95% CI)	
					Unadjusted	Adjusted
Main analysis^a^						
Lamotrigine or levetiracetam	1401	5.5 (2.7-9.3)	50	5.6	1.00 [Reference]	1.00 [Reference]
Valproate	961	10.6 (5.4-15.0)	67	6.7	0.99 (0.68-1.44)	1.02 (0.67-1.54)
Secondary analyses						
Excluding children of parents with malformations or NDDs^b^						
Lamotrigine or levetiracetam	1274	5.7 (2.8-9.5)	NA^c^	5.7	1.00 [Reference]	1.00 [Reference]
Valproate	902	11.1 (6.0-15.3)	NA^c^	6.6	0.97 (0.66-1.42)	0.99 (0.64-1.52)
Excluding children of mothers with epilepsy^d^						
Lamotrigine or levetiracetam	1373	5.5 (2.7-9.4)	50	5.7	1.00 [Reference]	1.00 [Reference]
Valproate	931	10.7 (5.5-15.2)	NA^c^	6.6	0.95 (0.65-1.39)	0.97 (0.64-1.49)
Excluding children with epilepsy^e^						
Lamotrigine or levetiracetam	1380	5.43 (2.6-9.2)	NA^c^	5.5	1.00 [Reference]	1.00 [Reference]
Valproate	924	10.5 (5.3-15.0)	59	6.2	0.93 (0.63-1.37)	0.98 (0.63-1.51)
Leaving out adjustment for maternal and paternal epilepsy						
Lamotrigine or levetiracetam	1401	5.5 (2.7-9.3)	50	5.6	NA	1.00 [Reference]
Valproate	961	10.6 (5.4-15.0)	67	6.7	NA	0.99 (0.65-1.49)
Leaving out adjustment for maternal and paternal education						
Lamotrigine or levetiracetam	1401	5.5 (2.7-9.3)	50	5.6	NA	1.00 [Reference]
Valproate	961	10.6 (5.4-15.0)	67	6.7	NA	1.11 (0.73-1.67)
Ending follow-up at 12 y						
Lamotrigine or levetiracetam	1401	5.5 (2.7-9.3)	NA^c^	5.8	1.00 [Reference]	1.00 [Reference]
Valproate	961	10.6 (5.4-11.0)	45	5.7	0.84 (0.56-1.27)	0.85 (0.53-1.36)

Abbreviations: HR, hazard ratio; IR, incidence rate; NA, not applicable; NDD, neurodevelopmental disorder.

^a^ Analysis using Cox proportional hazards regression comparing children of fathers exposed to valproate in monotherapy with children of fathers exposed to lamotrigine or levetiracetam in monotherapy, with unadjusted HRs and HRs adjusting for sex of the child, year of birth, paternal and maternal characteristics (age, psychiatric diagnoses, psychotropic medication use, epilepsy diagnosis, and educational level), and maternal valproate use during pregnancy.

^b^ Children were excluded if the parents had an NDD or congenital malformation diagnosis before 120 days before the first day of the last menstrual period.

^c^ Numbers are not shown because the differences from the numbers in the main analysis are less than 5.

^d^ Children were excluded if the mother had an epilepsy diagnosis before 120 days before the first day of the last menstrual period.

^e^ Children were excluded if they received an epilepsy diagnosis or had filled a prescription for antiseizure medication at any point during follow-up.

## Discussion

Despite our additional alignments of the study population and definitions to the IQVIA study report,^2^ this cohort study was unable to replicate the signal for NDDs in children with paternal valproate exposure. Instead, findings from these additional analyses are in line with our initial study^3^ and the conclusions of a recent systematic review,^6^ suggesting that paternal exposure to antiseizure medication in association with conception is unlikely to pose any major risk for the offspring. Although our study was large, it has limitations inherent to observational investigations based on register data.^3^ Given the potentially substantial implications of the EMA recommendation^1^ and the lack of reproducibility of the signal from the IQVIA study,^2^ we strongly urge for full transparency, including publication of the analytical code and full peer-review of the IQVIA study as soon as possible.

## Notes

Supplement 1. **eTable 1.** Variable Definitions **eTable 2.** Outcome Definitions in Children Diagnosed With Neurodevelopmental Disorders According to *ICD-10* and Filling for Prescriptions for Attention-Deficit/Hyperactivity Disorder (ADHD) Medication According to Anatomical Therapeutic Chemical Code (ATC Codes) **eTable 3.** Diagnostic Classification of Underlying Causes of Epilepsy (“Secondary Epilepsy”) According to *ICD-10* and Equivalent *ICD-8* Diagnoses **eTable 4.** *ICD-10* Codes Used for the Identification of Major Congenital Malformations **eTable 5.** Additional Description for Table 1
eReferences.

Supplement 2.
Data Sharing Statement
