# Supplementary material for: Risk of Neurodevelopmental Disorders and Paternal Use of Valproate During Spermatogenesis
Source: JAMA Netw Open. 2025 May 22;8(5):e2512139. doi: 10.1001/jamanetworkopen.2025.12139 (PMC12100447; doi:10.1001/jamanetworkopen.2025.12139)
Supplement: Supplement 1. — eTable 1. Variable Definitions eTable 2. Outcome Definitions in Children Diagnosed With Neurodevelopmental Disorders According to ICD-10 and Filling for Prescriptions for Attention-Deficit/Hyperactivity Disorder (ADHD) Medication According to Anatomical Therapeutic Chemical Code (ATC Codes) eTable 3. Diagnostic Classification of Underlying Causes of Epilepsy (“Secondary Epilepsy”) According to ICD-10 and Equivalent ICD-8 Diagnoses eTable 4. ICD-10 Codes Used for the Identification of Major Congenital Malformations eTable 5. Additional Description for Table 1 eReferences. [file jamanetwopen-e2512139-s001.pdf]

Supplemental Online Content

Christensen J, Trabjerg B, Werenberg Dreier J. Risk of neurodevelopmental disofrders and paternal use of valproate during spermatogenesis. *JAMA Netw. Open*. 2025;8(5):e2512139. doi:10.1001/jamanetworkopen.2025.12139

eTable 1. Variable Definitions

eTable 2. Outcome Definitions in Children Diagnosed With Neurodevelopmental Disorders According to *ICD-10* and Filling for Prescriptions for Attention-Deficit/Hyperactivity Disorder (ADHD) Medication According to Anatomical Therapeutic Chemical Code (ATC Codes)

eTable 3. Diagnostic Classification of Underlying Causes of Epilepsy ("Secondary Epilepsy") According to *ICD-10* and Equivalent *ICD-8* Diagnoses

eTable 4. *ICD-10* Codes Used for the Identification of Major Congenital Malformations

eTable 5. Additional Description for Table 1.

eReferences.

This supplemental material has been provided by the authors to give readers additional information about their work.

© 2025 Christensen J et al. *JAMA Network Open*.

eTable 1. Variable Definitions

The dataset contains all singleton children born alive in Denmark between 1997-2017 identified from the Medical Birth Register,[1] excluding children born to mothers who had undergone in vitro fertilization (in the period from the first day of the last menstrual period (LMP) minus 120 days until LMP plus 28 days), children with unknown or unrealistic gestational age at birth (gestational age <= 154 days and gestational age >315 days), children without a link to the father, children that died or emigrated before age 1 year, and children where the father did not fill a prescription for either valproate (Anatomical Therapeutic Chemical (ATC) Classification code (ATC code): N03AG01), lamotrigine (ATC code: N03AX09) or levetiracetam (ATC code: N03AX14) during the period LMP minus 120 days until LMP plus 14 days.

Variables for main adjustment: The adjusted estimates are adjusted for sex of the child, year of birth, paternal and maternal characteristics (age, psychiatric diagnoses, psychotropic medication use, epilepsy diagnosis, and educational level) and maternal valproate use during pregnancy.

Sex of the child, year of birth: Information from the Danish Civil Registration, birth year categorized in 1-year groups.

Paternal and maternal age: Age at time of childbirth (<25, 25-<35, 35+ years), specified separately for each parent.

Paternal and maternal psychiatric diagnoses: A history of psychiatric diagnoses given before LMP minus 120 days, specified separately for each parent. Diagnoses from the Danish Psychiatric Central Research Register using primary and secondary diagnoses (removing referral diagnoses) with ICD-10 codes F00-F99 and equivalent ICD-8 codes 290-315 for Mental and behavioral disorders (data available from 1969, outpatient and emergency from 1995). Yes/no-variables.

Paternal and maternal psychotropic medication use: Filling of prescriptions for psychotropic medication during LMP minus 120 days to LMP plus 14 days specified separately for each parent. Using prescriptions with ATC codes N05A (antipsychotics), N05B (anxiolytics) (excl. N05BA09 (clobazam)), N06A (antidepressants), N06BA01 (amfetamine), N06BA02 (dexamfetamine), N06BA04 (methylphenidate), N06BA09 (atomoxetine), N06BA11 (dexmethylphenidate), N06BA12 (lisdexamfetamine). Yes/no-variables.

Paternal and maternal epilepsy diagnoses: An epilepsy diagnosis given before LMP minus 120 days, specified separately for each parent. Diagnosis from the Danish National Patient Register using primary and secondary diagnoses (removing referral diagnoses) with ICD-10 code G40.x and equivalent ICD-8 codes 345 (excl. 345.29) for epilepsy (data available from 1977, outpatient and emergency admissions from 1995). Yes/no-variables.

Paternal and maternal educational level: highest completed educational level in the year of LMP minus 120 days, specified separately for each parent. A four-category variable (primary education or missing, high school or vocational education, short cycle higher education or bachelor, master or PhD).

Maternal valproate use during pregnancy: filling of prescriptions for valproate (ATC code N03AG01) during LMP minus 30 days to delivery.

Other variables:

Estimated paternal valproate dose: Average daily dose calculated as the sum of the defined daily doses from all valproate prescriptions filled in the period LMP minus 120 to LMP plus 14 divided by the number of days in the period. (high: >750 mg/day, low: ≤750 mg/day i.e. using half of the Defined Daily Dose (DDD) for valproate as the cutoff).

Paternal epilepsy of unknown etiology: An epilepsy diagnosis given before LMP minus 120 days, where the first epilepsy diagnosis was not preceded by an underlying symptomatic cause for epilepsy (diagnoses given in supplemental Table 2).

Parental NDD or congenital malformations: A history of NDD or congenital malformation diagnoses given before LMP minus 120 days for any of the parents. Diagnoses from the Danish National Patient Register using primary and secondary diagnoses (removing referral diagnoses) were used to identify NDD (Intellectual Disability, ICD-10: F70-79, Autism Spectrum Disorders, ICD-10: F84 (excl F84.2-F84.4), ADHD, ICD-10: F90.0+F98.8, Disorders of Psychological

© 2025 Christensen J et al. *JAMA Network Open*.

Development, ICD-10: F80-83) and congenital malformations: Q00-99, D181A, D215, D821, P350, P351, P371 (excluding minor malformations, see Supplemental Table 3)

Childhood epilepsy or use of antiseizure medication: A diagnosis of epilepsy or a prescription of antiseizure medication filled at any point in time during follow up (i.e. before death, emigration, a first diagnosis of NDD, or the end of follow-up (December 31, 2018)). Diagnoses from the Danish National Patient Register using primary and secondary diagnoses (removing referral diagnoses) with ICD-10 code G40.x for epilepsy. Prescriptions from the Danish Prescription Register with ATC N03A (antiepileptics) or N05BA09 (clobazam).

© 2025 Christensen J et al. *JAMA Network Open*.

**eTable 2.** Outcome Definitions in Children Diagnosed with Neurodevelopmental Disorders According to International Statistical Classification of Diseases and Related Health Problems, Tenth Revision (ICD-10) codes	
**Neurodevelopmental disorders**	**ICD-10 codes**
Intellectual disability	F70-79
Disorders of psychological development	F80-83
Autism spectrum disorder	F84 [excluding codes F84.2-F84.4]
Attention-deficit/hyperactivity disorder (ADHD)	F90.0 and F98.8
In secondary analyses, we included filling of prescriptions for Attention-deficit/hyperactivity disorder (ADHD) medication according to Anatomical Therapeutic Chemical Codes (ATC Codes): N06BA01 (amfetamine), N06BA02 (dexamfetamine), N06BA04 (methylphenidate), N06BA09 (atomoxetine), N06BA12 (lisdexamfetamine) N02CX02 (clonidine)	

© 2025 Christensen J et al. *JAMA Network Open*.

eTable 3. Diagnostic Classification of Underlying Causes of Epilepsy ("Secondary Epilepsy") According to *ICD-10* and Equivalent *ICD-8* Diagnoses

**Disease**		**ICD-8 codes**	**ICD-10 codes**
Infections	CNS infections	013, 027.01, 036.09, 040-043, 045- 046, 052.01, 053.02, 054.03, 055.01, 056.01, 062-065, 071.99, 072.02, 075.01, 079.29, 090.49, 094.9, 320, 322-324, 474	A02.2C, A06.6, A17, A22.9C, A32.1, A39.0, A50.4, A51.4B, A52.1A-B, A54.8A, A54.8D, A80-89, B00.3-00.4, B01.0-01.1, B02.0-02.1, B05.0-05.1, B06.0, B26.1-26.2, B37.5, B45.1, B58.2, B60.2, E23.6A, G00-09
	Tetanus	037.99, 670.04	A33-35
	Malaria	084	B50-54
	Cysticercosis	123.19	B69.0
	Cystic echinococcis	122.89, 122.99	B67.3-67.4, B67.8-67.9
Conditions originating in the perinatal period.		760-779	P00-P96
Malformations		740-759	Q00-Q99
Brain tumors		191, 198.39, 198.40, 198.41, 225	C70-72, C79.3 D32-D33
Traumatic brain injury		850.99, 800.99-801.09, 803.99, 851.29-854.99	S06.0, S02.0-S02.1, S02.7, S02.9, S06.1-S06.9
Stroke		430-438	I60-69

We classified fathers' epilepsy into epilepsy with underlying etiology ("secondary epilepsy") if the first epilepsy diagnosis was preceded by any of the following conditions: CNS infections, traumatic brain injury, stroke, brain neoplasm, perinatal complications, or congenital malformations, and epilepsy with unknown underlying etiology ("idiopathic epilepsy") if the first epilepsy diagnosis was not preceded by any of these conditions.[2]

© 2025 Christensen J et al. *JAMA Network Open*.

**eTable 4.** *ICD-10* Codes Used for the Identification of Major Congenital Malformations		
	**Included codes (incl subgroups)**	**Excluded minor malformations**
All major malformations	Q00-99, D181A, D215, D821, P350, P351, P371	Q078D, Q078G, Q101-Q105, Q135, Q170-Q175, Q179-Q182, Q184-Q187, Q189, Q211C, Q246, Q254E, Q261, Q314, Q318H, Q320, Q322, Q331, Q357, Q381, Q382, Q385B, Q400, Q401, Q430, Q444, Q458B, Q501, Q502, Q505, Q523, Q525, Q527, Q53, Q544, Q552F, Q552B, Q610, Q627, Q633, Q653-Q656, Q658, Q659, Q661- Q669, Q670-Q675, Q678, Q680, Q682A, Q683, Q684, Q685, Q740G, Q752, Q753, Q760, Q764L, Q765, Q766A-Q766C, Q767C, Q825, Q833, Q845, Q846, Q899, Q95

© 2025 Christensen J et al. *JAMA Network Open*.

eTable 5. Additional Description for Table 1

Main analysis, composite endpoint of all neurodevelopmental disorders (NDDs).
HRs with 95% CIs of NDD (composite endpoint of intellectual disability, disorders of psychological development, autism spectrum disorder and ADHD) using Cox Proportional Hazards regression comparing children of fathers exposed to valproate in monotherapy with children of fathers exposed to lamotrigine or levetiracetam in monotherapy, unadjusted and adjusting for sex of the child, year of birth, paternal and maternal characteristics (age, psychiatric diagnoses, psychotropic medication use, epilepsy diagnosis, and educational level) and maternal valproate use during pregnancy.
In the sensitivity analysis with a restricted exposure window from 60 days before the first day of the last menstrual period (LMP) to 14 days after the LMP, paternal valproate exposure was not associated with an increased risk of NDDs (composite end point, adjusted HR, 0.96 (95% CI, 0.62-1.49) (exposed/NDD cases; valproate: 824/56, lamotrigine/levetiracetam: 1254/NA).
In analyses with follow-up time split in two 2 periods (age 1 to 7.5 years and 7.5 years to end of the study), paternal valproate exposure was not associated with an increased risk of NDDs in either the early (adjusted HR = 1.30 (95% CI: 0.66-2.57), exposed/NDD cases; valproate: 961/23, lamotrigine/levetiracetam: 1401/22) or late (adjusted HR, 0.89; 95% CI, 0.52-1.50), exposed/NDD cases; valproate: 682/44, lamotrigine/levetiracetam: 594/28) follow-up period.
Intellectual disability
HRs with 95% CIs of intellectual disability (International Statistical Classification of Diseases and Related Health Problems, Tenth Revision (ICD-10) codes: F70-F79).
Disorders of psychological development
HRs with 95% CIs of disorders of psychological development (ICD-10 codes: F80-F83).
Autism spectrum disorder
HRs with 95% CIs of autism spectrum disorder (ICD-10 code: F84 (excluding F84.2-F84.4)).
ADHD (attention-deficit/hyperactivity disorder)
HRs with 95% CIs of ADHD (ICD-10 codes: F90.0 and F98.8).

Secondary analyses
Valproate dose
HRs with 95% CIs of NDD (composite endpoint). Children of fathers exposed to a high dose and low dose of valproate in monotherapy compared with children of fathers exposed to lamotrigine or levetiracetam in monotherapy.
Accounting for calendar trends (matched on birth year)
HRs with 95% CIs of NDD (composite endpoint). Children of fathers exposed to valproate in monotherapy compared with children of fathers exposed to lamotrigine or levetiracetam in monotherapy matched on birth year, resulting in equal number of exposed and unexposed children per birth year.
Allowing for polytherapy and monotherapy in both groups
HRs with 95% CIs of NDD (composite endpoint). Children of fathers exposed to valproate compared with children of fathers exposed to lamotrigine or levetiracetam, allowing both monotherapy and polytherapy in both groups. Children where the father filled prescriptions for valproate in combination with lamotrigine and/or levetiracetam were only included in the valproate group.
Including ADHD medication in definition of NDD
HRs with 95% CIs of NDD (composite endpoint, including ADHD medication (Medication used for childhood attention-deficit/hyperactivity disorder: N06BA01 (amfetamine), N06BA02 (dexamfetamine), N06BA04 (methylphenidate), N06BA09 (atomoxetine), N06BA12 (lisdexamfetamine) N02CX02 (clonidine)).
Restricted to fathers with epilepsy
HRs with 95% CIs of NDD (composite endpoint). Children of fathers with epilepsy exposed to valproate in monotherapy compared with children of fathers with epilepsy exposed to lamotrigine or levetiracetam in monotherapy.
Restricted to fathers with epilepsy, matched on birth year
HRs with 95% CIs of NDD (composite endpoint). Children of fathers with epilepsy exposed to valproate in monotherapy compared with children of fathers with epilepsy exposed to lamotrigine or levetiracetam in monotherapy matched on birth year, resulting in equal number of exposed and unexposed children per birth year.
Restricted to fathers with epilepsy of unknown underlying cause
HRs with 95% CIs of NDD (composite endpoint). Children of fathers with epilepsy of unknown underlying cause exposed to valproate in monotherapy compared with children of fathers with epilepsy of unknown underlying cause exposed to lamotrigine or levetiracetam in monotherapy.
Restricted to fathers with epilepsy of unknown underlying cause, matched on birth year
HRs with 95% CIs of NDD (composite endpoint). Children of fathers with epilepsy of unknown underlying cause exposed to valproate in monotherapy compared with children of fathers with epilepsy of unknown underlying cause exposed to lamotrigine or levetiracetam in monotherapy matched on birth year, resulting in equal number of exposed and unexposed children per birth year.

© 2025 Christensen J et al. *JAMA Network Open*.

eReferences

1. Bliddal M, Broe A, Pottegard A, Olsen J, Langhoff-Roos J. The Danish Medical Birth Register. *Eur J Epidemiol*. Jan 2018;33(1):27-36. doi:10.1007/s10654-018-0356-1
2. Christensen J, Trabjerg BB, Wagner RG, et al. Prevalence of epilepsy: a population-based cohort study in Denmark with comparison to Global Burden of Disease (GBD) prevalence estimates. *J Neurol Neurosurg Psychiatry*. Nov 27 2024;doi:10.1136/jnnp-2024-334547

© 2025 Christensen J et al. *JAMA Network Open*.
