# Supplementary material for: Risk of Neurodevelopmental Disorders and Paternal Use of Valproate During Spermatogenesis
Source: JAMA Netw Open. 2025 May 22;8(5):e2512139. doi: 10.1001/jamanetworkopen.2025.12139 (PMC12100447; doi:10.1001/jamanetworkopen.2025.12139)
Supplement: Supplement 2. — Data Sharing Statement [file jamanetwopen-e2512139-s002.pdf]

Data Sharing Statement

Christensen. Risk of Neurodevelopmental Disorders and Paternal Use of Valproate During Spermatogenesis. *JAMA Netw Open*. Published May 22, 2025.
doi:10.1001/jamanetworkopen.2025.12139

Data
Data available: No

Additional Information
Explanation for why data not available: Data were based on Danish national registers, and individual level data cannot be shared. However, summary statistics, in addition to the results provided in the results section and supplementary material, may be provided on request. The analytical code is published on GitHub with the following link: https://github.com/btrabjerg/The-paternal-valproate-project.
